# When and How to Evaluate Vitamin D Status? A Viewpoint from the Belgian Bone Club

**DOI:** 10.3390/nu16152388

**Published:** 2024-07-23

**Authors:** Bruno Lapauw, Michaël R. Laurent, Serge Rozenberg, Jean-Jacques Body, Olivier Bruyère, Evelien Gielen, Stefan Goemaere, Laura Iconaru, Etienne Cavalier

**Affiliations:** 1 Department of Endocrinology, Unit for Osteoporosis and Metabolic Bone Diseases, Ghent University Hospital, 9052 Ghent, Belgium; stefan.goemaere@uzgent.be; 2 Department of Internal Medicine and Pediatrics, Ghent University, 9052 Ghent, Belgium; 3 Geriatrics Department, Imelda Hospital, 2820 Bonheiden, Belgium; 4 Centre for Metabolic Bone Diseases, University Hospitals Leuven, 3000 Leuven, Belgium; evelien.gielen@uzleuven.be; 5 Department of Obstetrics and Gynecology, CHU St Pierre, Brussels & Université Libre de Bruxelles, 1000 Bruxelles, Belgium; serge.rozenberg@stpierre-bru.be; 6 Department of Medicine, CHU Brugmann, Université Libre de Bruxelles, 1020 Brussels, Belgium; jean-jacques.body@chu-brugmann.be (J.-J.B.); laura.iconaru@chu-brugmann.be (L.I.); 7 WHO Collaborating Center for Public Health Aspects of Musculoskeletal Health and Ageing, Research Unit in Public Health, Epidemiology and Health Economics, Department of Sport and Rehabilitation Sciences, University of Liège, 4000 Liège, Belgium; olivier.bruyere@uliege.be; 8 Geriatrics & Gerontology, Department of Public Health and Primary Care, KU Leuven, 3000 Leuven, Belgium; 9 Department of Geriatric Medicine, University Hospitals Leuven, 3000 Leuven, Belgium; 10 Department of Clinical Chemistry, University of Liège, CIRM, CHU de Liège, 4000 Liège, Belgium; etienne.cavalier@chuliege.be

**Keywords:** vitamin D, screening guidelines, cost-effectiveness

## Abstract

Low serum vitamin D levels have been associated with a variety of health conditions which has led the medical community but also the general population to evaluate vitamin D status quite liberally. Nevertheless, there remain questions about the efficacy and cost-effectiveness of such a broad and untargeted approach. This review therefore aims to summarize the current evidence and recommendations on when and how to evaluate vitamin D status in human health and disease. For the general population, most guidelines do not recommend universal screening but suggest a targeted approach in populations at risk. Also, some guidelines do not even recommend evaluating vitamin D status when vitamin D substitution is indicated anyway, such as in children or patients receiving anti-osteoporosis drugs. In those guidelines that recommend the screening of vitamin D status, serum 25(OH)D levels are universally proposed as the preferred screening tool. However, little attention is given to analytical considerations and almost no guidelines discuss the timing and frequency of screening. Finally, there is the known variability in diagnostic thresholds for defining vitamin D insufficiency and deficiency. Overall, the existing guidelines on the evaluation of vitamin D status differ broadly in screening strategy and screening implementation, and none of these guidelines discusses alternative screening modes, for instance, the vitamin metabolic ratio. Efforts to harmonize these different guidelines are needed to enhance their efficacy and cost-effectiveness.

## 1. Introduction

Vitamin D is important for numerous biological processes, from intestinal calcium uptake to innate immune responses. Several guidelines recommend the measurement of 25-hydroxyvitamin D (25(OH)D) to assess an individual’s vitamin D status, and the results of these measurements are interpreted against cut-offs which define if the patient is vitamin D ‘deficient’ or not. Unfortunately, these cut-offs may vary from guideline to guideline, and there is no universal consensus among the experts [1]. Nevertheless, the most commonly suggested 25(OH)D cut-off to define vitamin D sufficiency, particularly in the general population, is set at 50 nmol/L (20 ng/mL) [2]. This cut-off has also been suggested to be used in Belgium by the Belgian Bone Club (BBC) in the Guidelines published in 2020 [3] and was thus used in this study to define vitamin D deficiency unless specified otherwise. Of note, the BBC has also provided an upper limit of safety for 25(OH)D at 125 nmol/L (50 ng/mL), even if the literature does not align with this cut-off. Anyway, whatever the cut-off used, severe vitamin D deficiency or disorders in vitamin D metabolism can lead to a variety of pathophysiological processes, and optimal exposure to vitamin D is important during every stage of life, especially but not exclusively for the musculoskeletal system [4,5]. During the recent decades, reports on associations between vitamin D and a myriad of non-skeletal disorders received much attention. This has led to the pandemic testing of ‘vitamin D status’ [6,7] and physicians and other healthcare workers advising on supplementary vitamin D intake as a new sort of *panacea*. Although these practices might reflect a life course approach [8], currently, there remains insufficient evidence that vitamin D supplementation is effective in a healthy, vitamin D-replete adult population [9,10,11,12]. Also, from a health economics perspective, untargeted vitamin D screening seems not to be cost-effective. In Belgium, the public health insurance RIZIV/INAMI reimbursed EUR 13.8 million for 25(OH)D immunoassay tests in 2019 which has consequently led this body to restrict the number of reimbursed tests to one per year for the general population. In an analysis of more than 4 million Medicare-funded vitamin D tests performed in 2020 in Australia, it was estimated that more than three quarters did not provide a health benefit, resulting in more than AUD 87 million as unnecessary test costs [13]. However, studies adequately investigating the cost-effectiveness of vitamin D screening in adults are lacking. To properly identify individuals that might benefit from vitamin D suppletion or other interventions, better guidance is needed in whom and when to screen for vitamin D deficiency or vitamin D-related disorders. Equally important are some analytical challenges that remain when appraising vitamin D status and metabolism. This narrative review, made by the Belgian Bone Club, a leading scientific society active in the field of bone health for more than 30 years, will summarize the current evidence and recommendations on when and how to evaluate vitamin D status in human health and disease. We will discuss both clinical indications and laboratory aspects, addressing potential pitfalls, areas of uncertainty, and limitations.

### 1.1. Vitamin D Metabolism

#### Vitamin D—What’s in a Name?

Vitamin D is commonly used as a generic term encompassing various biologically active and inactive metabolites. Although somewhat confusing, this is understandable as vitamin D metabolism is complex, and around 50 different vitamin D metabolites have been described [14,15]. Contrary to the conventional definition, vitamin D is not strictly a ‘true’ vitamin, as it can be synthesized by the skin and functions as a (pro)hormone, undergoing transformation within the body into active and inactive metabolites. In humans, ergocalciferol (vitamin D2, derived from plant and yeast intake) and cholecalciferol (vitamin D3, derived from animal intake and the endogenous conversion of 7-dehydrocholesterol by UVB radiation in the skin) are converted to 25(OH)D2 and 25(OH)D3 by the 25-hydroxylase enzyme in the liver. This enzymatic conversion is poorly regulated, and therefore, 25(OH)D levels are dependent on the availability of vitamin D2 and D3. 25(OH)D is then further converted by 1-alpha hydroxylase to calcitriol (1α,25(OH)_2_D), a process which mostly occurs in the kidney, although this enzyme is also expressed in other tissues. Calcitriol is considered the most biologically active metabolite and can bind to the vitamin D receptor (VDR) with high affinity. Therefore, renal 1α-hydroxylase activity is strictly regulated by parathyroid hormone (PTH), fibroblast growth factor 23 (FGF23), calcium, phosphate, and calcitriol levels. 25(OH)D can also be converted to metabolites such as 3-epi-25(OH)D and 24,25(OH)_2_D. But the conversion of 25(OH)D and 1α,25(OH)_2_D by 24-hydroxylase into 24,25(OH)_2_D and 1α,24,25(OH)_2_D, respectively, especially seems critical in controlling the levels of active vitamin D. Although 24-hydroxylase activity is considered as the first step in vitamin D catabolism, preclinical data suggest that there might be a physiological role for 24,25(OH)_2_D in fracture repair via interaction with a specific receptor [5]. The physiological functions of C3 epimeric metabolites, such as 3-epi-25(OH)D and 3-epi-1α,25(OH)D_2_, and those of metabolites such as 1β,25(OH)_2_D remain undetermined [15,16]. Further, cytochrome P450 (CYP) enzyme 3A4 (CYP3A4) can also oxidize 25(OH)D and 1,25(OH)D2 into 4β-hydroxylated, inactivated substrates, as shown in patients with rickets due to CYP3A4-activating mutations [17].

### 1.2. Determinants of Vitamin D Status

Many studies have investigated the determinants of vitamin D status, revealing complex interactions between genetic predisposition, environmental factors, lifestyle choices, and dietary behaviour. One large study confirmed that factors such as female sex, older age, obesity, geographical location at latitudes further from the equator, physical inactivity and sedentarism, limited sun exposure, and certain genetic mutations are associated with lower vitamin D levels [18]. Smoking and alcohol consumption have also been associated with vitamin D insufficiency [19,20]. Notably, dietary intake played a minor role, emphasising the importance of lifestyle and environmental factors. An interesting review of the literature highlighted the crucial role of sunlight exposure in vitamin D synthesis and found that factors such as age, skin type, clothing choices, and other lifestyle factors determining sun exposure significantly affect vitamin D production [21]. Seasonal variations in vitamin D levels were also observed, again illustrating the importance of sunlight exposure. Regarding the effect of genetics, several studies showed and confirmed that specific genetic variations, such as those near the genes encoding vitamin D-binding protein (*GC*), 7-dehydrocholesterol reductase (*DHCR7*), and enzymes, such as 25-hydroxylase (*CYP2R1*) and 24-hydroxylase (*CYP24A1*), have been identified as influential factors in vitamin D metabolism [22,23]. Although these genetic factors play a role, and ethnicity should probably be considered in evaluating vitamin D status as differences in total serum 25(OH)D and vitamin D-binding protein levels have been described [24,25], their overall contribution to vitamin D status at the individual level appears to be relatively modest compared with environmental factors. The latter is relevant, as it might contribute to the development of patient education strategies pertaining to nutritional choices and leisure time activities.

Almost all severe chronic diseases have been associated with lower vitamin D levels [26], owing to different mechanisms (Table 1). Although some specific mechanisms are clearly recognized in some diseases, the aetiology of vitamin D deficiency in most patients with chronic disease is multifactorial and involves both disease-related and -independent mechanisms (e.g., obesity in a patient with CKD and limited sun exposure).

**Table 1:** Different mechanisms of vitamin D deficiency in chronic diseases and conditions.

Mechanism	Example Conditions
Reduced sunshine exposure from impaired outdoor physical activity	Chronic fatigue syndrome, fibromyalgia COPD Depression Dementia Heart failure Neuromuscular diseases Osteoarthritis Parkinson’s disease Rheumatic conditions
Reduced sunshine exposure from avoidance of skin exposure to UV light	Dermatitis Melanoma/non-melanoma skin cancer Porphyria Psoriasis Systemic lupus erythematosus Xeroderma pigmentosum
Increased body fat	Metabolic syndrome Obesity Type 2 diabetes
Malabsorption and/or gastrointestinal loss of fat-soluble vitamins	Bariatric surgery Cystic fibrosis Exocrine pancreatic insufficiency Inflammatory bowel diseases Nutritional and eating disorders Primary biliary cirrhosis/primary sclerosing cholangitis Short bowel syndrome
Renal loss of vitamin D-binding protein	Chronic kidney disease with proteinuria
Impaired vitamin D synthesis	Old age (reduced vitamin D synthesis in the skin) Cirrhosis, fatty liver disease/metabolic syndrome (25-hydroxylation) Chronic kidney disease (impaired 1-alpha hydroxylation)
Increased catabolism	CYP3A4 enzyme-inducing medications (for tuberculosis, epilepsy, etc.) CYP3A4-activating mutations

In clinical populations, vitamin D deficiency has been associated with infectious diseases, malignancies, psychiatric disorders, and cardiac, pulmonary, urological, renal, and metabolic disorders [27]. Observational studies have reported links between vitamin D deficiency and outcomes in cancers, cardiovascular diseases, dementia, depression and psychiatric disorders, type 2 diabetes and metabolic syndrome, tuberculosis and respiratory tract infections, autoimmune diseases, prematurity, chronic kidney disease, and osteoporosis and fracture patients [26]. Importantly, in most patient cohort studies, demographic determinants like age, body weight, sunshine exposure, skin pigmentation or veiling, etc. were still key determinants of vitamin D status.

CYP3A4-inducing drugs can cause vitamin D deficiency. Conversely, vitamin D induces CYP3A4 [28]. CYP3A4 inducers can even be used to treat vitamin D-mediated hypercalcemia [29]. Strong inducers include anti-epileptic drugs (carbamazepine, phenytoin, phenobarbital, primidone, high-dose topiramate), cancer drugs (apalutamide, enzalutamide, dabrafenib, mitotane, vemurafenib), certain antibiotic/tuberculostatic drugs (rifampicin, rifabutin), and some traditional medicines (notably, St John’s wort, *Hypericum perforatum*). Although some studies suggest that glucocorticoids induce vitamin D degradation, the association between glucocorticoid use and vitamin D deficiency appears to be mainly mediated by disease status [30]. Taken together, screening for vitamin D deficiency is appropriate in many chronic conditions and in patients treated with strong CYP3A4-inducing drugs. Guidelines should consider including 25(OH)D measurements at baseline and at least annually in disease-monitoring programs, especially in at-risk individuals with additional risk factors (e.g., based on older age, obesity, lower sunshine exposure, etc.).

Hypervitaminosis D is considered as a clinical condition characterized by severe hypercalcemia rather than defined by serum 25(OH)D levels [31]. When not resulting from overdosing, hypervitaminosis D is usually associated with granulomatous diseases with increased 1α-hydroxylation (e.g., sarcoidosis, tuberculosis, lymphoma, fungal diseases, leprosy, berylliosis, etc.), which may lead to vitamin D-mediated hypercalcemia, hypercalciuria, bone loss, and kidney stone formation. In case of monoallelic or biallelic *CYP24A1* mutations, modest-to-severe vitamin D excess with kidney stone formation and/or hypercalcemia may also occur [32]. Rarely, autosomal-recessive mutations in *SLC34A1* may cause primary renal phosphate wasting, the downregulation of FGF23, inappropriate elevations in 1,25-dihydroxyvitamin D levels, and idiopathic infantile hypercalcemia [33].

## 2. Appraisal of Vitamin D Status

### 2.1. In Whom and When?

In the next paragraphs, we will discuss determinants and importance of vitamin D status in different periods of life and clinical conditions. An overview of guideline recommendations is given in Table 2.

#### 2.1.1. During Childhood and Youth

Inadequate vitamin D levels occur not only in adults but also in infants, children, and adolescents [34]. Previous research has highlighted the importance of maintaining adequate serum vitamin D concentrations to support proper growth, plate calcification, and bone mineralisation. Evidence suggests that serum 25(OH)D3 levels below 20–25 nmol/L over a prolonged period may lead to the development of rickets and osteomalacia [35]. Various risk factors in children, such as seasonal variations (mainly winter), limited outdoor time and thus sunlight exposure, non-white ethnicity, advanced pubertal stage, limited milk intake, lower socioeconomic status, and female gender, alongside modern lifestyle shifts like the global surge in childhood and adolescent obesity, contribute significantly to the prevalence of vitamin D insufficiency or deficiency [34,36]. As a result, many national and international health organisations recommend universal vitamin D suppletion in all infants and children till 18 years of age. As such, also because the precise thresholds defining subclinical vitamin D deficiency in children remain unclear, the current guidelines do not recommend universal screening in children. Instead, screening for vitamin D deficiency should only be performed in at-risk groups such as in children with growth issues, those with obesity, children with dark skin, children living at higher latitudes, those receiving chronic anticonvulsant or glucocorticoid treatment, and those suffering from malabsorption, calcium or phosphate disorders, or having skeletal disorders [37,38,39,40,41].

#### 2.1.2. During Pregnancy and Lactation

Pregnancy is a physiologically demanding life period during which the maternal body undergoes numerous changes to support the growth and development of the foetus. Adequate nutrition, including sufficient minerals and vitamins, is essential for maintaining maternal health and promoting optimal foetal development. Vitamin D deficiency is estimated to affect 40–98% of pregnant individuals worldwide [42]. Low serum 25(OH)D levels have been associated with poor maternal and neonatal health outcomes, but it is unclear whether poor health is caused or worsened by low vitamin D levels, or whether low vitamin D levels are surrogate markers of poor health. For instance, a systematic review and meta-analysis of 31 studies found that serum 25(OH)D levels of <75 nmol/L were associated with a 50% increased gestational diabetes risk and an 80% increased pre-eclampsia risk, and that serum 25(OH)D levels of <37.5 nmol/L were associated with an 85% increased risk for small for gestational age infants (SGA) [43]. Nevertheless, trials assessing vitamin D supplementation have failed to report consistent results. A systematic review of 24 randomized clinical trials comprising 5405 participants found that vitamin D supplementation during pregnancy was associated with a 30% lower risk of SGA but did not affect foetal or neonatal mortality or congenital abnormalities [44]. Neonates with prenatal vitamin D supplementation also had higher 25(OH)D levels, higher birth weight, and higher body weight at the age of one year, and sub-analyses reported a reduction in neonatal morbidity. From a 2019 Cochrane review, it was concluded that vitamin D suppletion in pregnant women probably reduces the risk of pre-eclampsia, gestational diabetes, and low birthweight and may also reduce the risk of severe postpartum haemorrhage without affecting the risk of preterm birth [45]. However, there are also negative studies [46], and another systematic review concluded that most trials on prenatal vitamin D were small and of low quality, resulting in insufficient evidence to produce recommendations [47]. Given this, most professional societies do not recommend universal vitamin D deficiency screening in all pregnant women. However, in those women considered at risk for deficiency, measuring serum 25(OH)D concentrations can be considered [48]. If deficiency is suspected, women should be informed to only take vitamin supplements that are specific for pregnancy and lactation, since other multivitamin supplements may also contain vitamin A (retinol), which may be harmful to the foetus [49]. In contrast, however, the 2024 Clinical Practice Guideline of the Endocrine Society recommends empiric vitamin D supplementation in all pregnant women because of its potential to improve various maternal and foetal outcomes [41].

#### 2.1.3. During Menopause

The European Menopause and Andropause Society stresses the importance of optimal vitamin D exposure for skeletal health and possible non-skeletal effects during and after menopausal transition. Nevertheless, due to insufficient evidence, their recent consensus statement provides no guidance on which menopausal women to screen nor by what frequency screening should be performed [50].

#### 2.1.4. In Older Individuals

Vitamin D deficiency is very prevalent in older individuals, especially if institutionalized or home-bound, and associates with an increased risk of poor skeletal and extra-skeletal outcomes. Older individuals are at an increased risk of vitamin D deficiency due to reduced sun exposure, the reduced capacity of the skin to synthesize vitamin D3, and a lower production of 1,25(OH)2D associated with the age-related decline in renal function [51,52]. Ageing also leads to a decrease in the number of VDR in organ systems involved in calcium metabolism such as the intestine, leading to relative intestinal resistance to 1,25(OH)2D and lower calcium absorption [51]. Beyond that, age-related increases in fat mass may contribute to lower circulating levels of 25(OH)D, as do comorbidities and drugs (see Table 1) [51]. However, the observation that mean serum 25(OH)D is >50 nmol/L in adults aged >70 years in the 2005–2006 National Health and Nutrition Examination Survey and that 25(OH)D in several groups in Amsterdam measured with the same assay gradually declines from healthy adults over independent older individuals to institutionalized older persons and patients with hip fractures indicates that not age per se but rather frailty status is an important determinant of 25(OH)D levels [53,54].

Several approaches could be taken regarding the appraisal of vitamin D in the older population. While a population-based screening for vitamin D deficiency is not recommended by guidelines such as the 2021 recommendation of the U.S. Preventive Services Task Force [55] and the 2024 Endocrine Society Clinical Practice guideline [41], the approach differs in persons at risk of vitamin D deficiency. For instance, the European Society of Clinical and Economical Aspects of Osteoporosis, Osteoarthritis and Musculoskeletal diseases recommends vitamin D supplementation for those at increased risk of deficiency, including persons at risk of osteoporosis, on concurrent osteoporosis treatment, or with a fragility fracture, as well as older people at risk of falling and subjects with limited sun exposure [56]. In its 2020 guideline for the management of postmenopausal osteoporosis, the Belgian Bone Club recommends vitamin D screening in postmenopausal women with at least one major risk factor for osteoporosis to undergo a further assessment for osteoporosis [3]. Since age ≥ 65 years is considered one of the major risk factors of osteoporosis, this implies that the systematic measurement of 25(OH)D should be performed in all older individuals. The rationale is to avoid supplementation in persons with normal serum levels, at low risk of fractures, and without pharmacological osteoporosis treatment [3]. The vitamin D megatrials have indeed shown that vitamin D supplementation in vitamin D-replete individuals does not provide any health benefit [56]. In older individuals with 25(OH)D ≤ 50 nmol/L or in older adults who start pharmacological treatment for osteoporosis, 800–1000 IU of vitamin D per day is recommended, with the monitoring of the 25(OH)D level [3]. Because of the substantial individual variation in 25(OH)D following supplementation, it is indeed recommended to retest serum 25(OH)D levels after about 3 months of supplementation to confirm that the target level has been reached [57,58]. However, in older adults with 25(OH)D > 50 nmol/L, who are at low fracture risk and do not receive pharmacological treatment for osteoporosis, vitamin D supplementation is not recommended. In these individuals, the measurement of 25(OH)D could be repeated every two years, which is the period recommended by the BBC guidelines to perform a new screening check for osteoporosis [3]. In contrast, the latest Endocrine Society Clinical Practice Guideline suggests against routine testing for serum 25(OH)D levels but instead suggests empiric supplementation in the general population aged 75 years and older. Neither do they suggest routine follow-up testing to guide vitamin D supplementation dosing [41].

#### 2.1.5. In Obesity and after Bariatric Surgery

A low body mass index (BMI) is associated with an increased fracture risk, but the risk of fragility fractures also increases in obesity [59,60]. The aetiology of higher fracture risk in these two extremes of body weight may be related to differences in bone quality, biomechanical disadvantages, hormonal influences, or factors related to a poor diet and/or reduced physical activity. Also, a high BMI was found to be associated with low serum 25(OH)D concentrations in several studies [61,62], and obese women have been shown to have higher PTH levels than their non-obese counterparts [63]. The most widely accepted hypothesis is that the fat-soluble vitamin D is more easily stored into fat cells (sequestration or volumetric dilution). However, other explanations have been proposed, including insufficient dietary intake, limited sunlight exposure, and lower hepatic synthesis [64]. Whether a poor vitamin D status contributes to the health consequences of overweight or obesity is not known, but several consequences of both conditions are overlapping [65]. Nevertheless, there is some epidemiological evidence suggesting that high vitamin D levels are associated with a lower incidence of obesity and diabetes [61]. These observations are, however, subject to several potential biases (e.g., people who are more physically active, and therefore at a lower risk of being overweight, and spend more time outdoors, and therefore have better vitamin D status). Results from intervention studies that assessed the value of vitamin D supplementation have not been encouraging in vitamin D-sufficient individuals. As in other conditions, it appears that any benefit of vitamin D for diabetes prevention, if present, is modest and does not pertain to a vitamin D-sufficient population [65]. It has also not been shown that a threshold higher than what is advised in non-obese individuals (i.e., 75 nmol/L) is of any benefit [66], although it should be noted that obese people need higher doses of vitamin D to reach the same threshold [67]. Given these multiple uncertainties, both the ESE and ES guidelines recommend against universal screening for vitamin D deficiency in adults with obesity [41,62].

However, clinically relevant vitamin D deficiency leading to secondary hyperparathyroidism and accelerated bone loss is observed in obese individuals after bariatric surgery and especially after malabsorptive procedures [68,69,70]. Moreover, the intestinal absorption of fat-soluble micronutrients, including vitamin D, is impaired after bariatric surgery along with that of calcium, even when the vitamin D status is optimal [71,72]. Further, because overall food consumption is dramatically decreased after surgery, smaller amounts of vitamin D-containing food may be consumed. As such, the general monitoring of serum 25(OH)D and PTH levels in patients after bariatric surgery is advised [62,73,74]. Also, response to supplementation after bariatric surgery is highly variable, so the periodic monitoring of supplementation is equally recommended [73,74,75].

#### 2.1.6. In Inflammatory Bowel Disease

Another indication where vitamin D status may be of concern is in patients with inflammatory bowel disease, in particular, Crohn’s disease (CD). Vitamin D deficiency is frequent in patients with CD owing to the combination of chronic inflammation, intestinal malabsorption, and lifestyle. Moreover, recent evidence provides a strong mechanistic basis for a role of vitamin D deficiency in the pathogenesis of CD complications [76,77]. Further, a meta-analysis of case–control and cross-sectional studies has shown that clinically diagnosed coeliac disease is also associated with an increase in fracture risk [78]. One proposed mechanism is that coeliac disease leads to dietary malabsorption and thus deficiency of vitamins and minerals, such as vitamin D and calcium [79]. Overall, clinical practice guidelines recommend monitoring for vitamin D deficiency in patients with inflammatory bowel disease, but the frequency of screening has not been defined [80,81].

#### 2.1.7. In Prevention of Fractures and in Addition to Anti-Osteoporosis Therapies

Vitamin D deficiency can lead to reduced bone mineral density (BMD), osteomalacia, high bone turnover, and fractures [52,82,83,84]. However, studies have reached mixed conclusions regarding the association between vitamin D levels and fracture incidence or the efficacy of anti-osteoporosis therapies. From a recent systematic review of meta-analyses, it was concluded that vitamin D substitution (together with calcium) might reduce fracture risk, but this finding was most likely driven by effects in institutionalized older persons [85]. On the other hand, vitamin D-replete adults with 25(OH)D levels in the range of 50–100 nmol/L are unlikely to benefit from vitamin D supplementation. Furthermore, vitamin D supplementation resulting in 25(OH)D levels above 100 nmol/L probably increases the risk of falls and fractures [56]. Confirming the importance of optimal vitamin D exposure to minimize fracture risk, two recent meta-analyses found that, compared to low serum 25(OH)D levels, high serum 25(OH)D levels protect against the risk of hip fractures in older patients [86,87]. As such, some [3,88,89,90], but not all [91,92,93,94,95,96], osteoporosis treatment guidelines recommend screening for vitamin D deficiency in patients at risk for osteoporosis and fragility fractures. In our last consensus paper, the BBC recommended to systematically assess and re-evaluate vitamin D status in all postmenopausal women with at least one major risk factor for osteoporosis who, therefore, underwent osteoporosis assessment. Such an approach allows to avoid the supplementation of vitamin D in those with normal serum 25(OH)D levels but also to evaluate the compliance of supplementation [3].

In postmenopausal women with osteoporosis, several studies suggested that adequate vitamin D repletion appears necessary to maximize the response to anti-osteoporosis treatment both in terms of BMD changes and anti-fracture efficacy [97,98,99]. In a real-life study, it was found that, in postmenopausal women treated with bisphosphonates, the odds of having a treatment response were larger in those women with a serum 25-OH D level of ≥75 nmol/L [100]. However, there is also evidence that baseline serum 25(OH)D level is less important for response to bisphosphate therapy when this is co-administered with cholecalciferol and calcium supplements [101]. Most professional societies recommend vitamin D supplementation for patients at a high risk of fracture and/or those receiving pharmacological osteoporosis treatments independent of baseline vitamin D status to avoid incident vitamin D deficiency and reduce the risk of hypocalcaemia [3,88,89,90,91,92,93,94,95]. However, except for the BBC’s advice for bi-annual re-evaluation [3], recommendations on the frequency of screening once an osteoporosis treatment is initiated are largely lacking.

#### 2.1.8. In Rheumatic Disorders

Low vitamin D levels have been reported in many different inflammatory rheumatic diseases and have been associated with disease activity [102]. These observations might not be so surprising given vitamin D’s role in regulating the immune system. Nevertheless, there is currently insufficient evidence that vitamin D therapy would significantly alter disease activity in rheumatic disorders [103,104,105], and active screening or monitoring of vitamin D status in these conditions is generally not recommended [106]. The situation changes when these patients are receiving glucocorticoid treatment, and although the evidence for calcium and vitamin D supplementation for fracture reduction in patients with rheumatic disorders needing glucocorticoid treatment is low to very low, supplementation thereof and monitoring of serum 25(OH)D levels are recommended by the American College of Rheumatology [107].

#### 2.1.9. In Chronic Kidney Disease (CKD)

In patients with CKD, as kidney function declines, disturbances in mineral and bone metabolism occur due to impaired vitamin D activation and increased PTH levels, resulting in CKD–mineral and bone disorder (CKD-MBD) [108]. Vitamin D deficiency or insufficiency is even more common in patients with CKD than in the general population and has been associated with increased mortality, rapid kidney function decline, and higher fracture risks in this population [109]. As such, the KDIGO guidelines and those of other societies recommend monitoring vitamin D status and the initiation of substitution in case of deficiency; however, the frequency of monitoring is not mentioned [108,109,110].

**Table 2:** Recommendations of different societies and organisations on evaluating vitamin D status.

Target Public	Scientific Body	Year	RDI	Screening Strategy	Screening Mode	Start of Screening	Screening Frequency	Diagnostic Thresholds
Children	ES [41]	2024	Not provided	Recommendation against routine testing	Serum 25(OH)D	NA	NA	NA
	French expert group [39]	2022	400–800 IU daily	Screening when there are signs of rickets	Serum total 25(OH)D	Not provided	Not provided	Insufficiency: <75 nmol/L Deficiency: <50 nmol/L
General population	IOF [111]	2024	800–1000 IU daily	Routine screening poorly justified	Serum 25(OH)D	NA	NA	NA
	ES [41]	2024	600 IU daily	Recommendation against routine testing	Serum 25(OH)D	NA	NA	NA
	USPSTF [55]	2021	NA	Not provided	Serum 25(OH)D	NA	NA	Not provided
	ESCEO [56]	2022	800–1000 IU daily	Not provided. Supplementation recommended in persons at increased risk of deficiency	Serum 25(OH)D	Not provided	Not provided	Insufficiency: <50 nmol/L Deficiency: <25 or 30 nmol/L
Pregnant women	ACOG [48]	2011	600 IU daily	To be considered in those at risk for deficiency	Serum 25(OH)D	Not provided	Not provided	Not provided
	ES [41]	2024	600 IU daily	Recommendation against routine testing	Serum 25(OH)D	NA	NA	NA
Postmenopausal women	BBC [3]	2020	800–1000 IU daily	Women with at least 1 major risk factor for osteoporosis who undergo assessment for osteoporosis	Serum 25(OH)D (accurate and standardized method)	Women with at least 1 major risk factor for osteoporosis	Monitoring in those on supplements; bi-annual rescreening in non-treated women	Treatment target > 50 nmol/L
	EMAS [50]	2023	800–2000 IU daily	To be considered in those at risk for deficiency	Serum 25(OH)D	Not provided	Not provided	Deficiency: <50 nmol/L Severe deficiency: <25 nmol/L
Obese adults	ESE [62]	2020	Not provided	Not routinely recommended	Serum 25(OH)D	Not provided	Not provided	Not provided
	ES [41]	2024	600 IU daily	Recommendation against routine testing	Serum 25(OH)D	NA	NA	NA
Inflammatory bowel disease	AGA [81]	2024	Not provided	All patients with inflammatory bowel disease	Serum 25(OH)D	Not provided	Not provided	Not provided
	BSG [80]	2019	Not provided	Adults with Crohn’s disease or ulcerative colitis	Serum 25(OH)D	Not provided	Not provided	Deficiency: <50 nmol/L
Rheumatic disorders	ACR [107]	2022	600–800 IU daily	Children and adults beginning or continuing chronic glucocorticoids at a dose of ≥2.5 mg/day for >3 months	Serum 25(OH)D	Before initiating treatment	Not provided	Target level 75 to 125 nmol/L
Chronic kidney disease	KDIGO [110]	2017	Not provided	Suggested in patients with CKD, especially when treated with antiresorptives	Serum 25(OH)D	Not provided	Not provided	Not provided, but should at least be 50–75 nmol/L
Older adults	IOF [57]	2010	800–1000 IU daily	Measure in those at risk for deficiency	Serum 25(OH)D	Not provided	Not provided; retest 3 months after supplementation	Insufficiency: <75 nmol/L Deficiency: <50 nmol/L
	ES [41]	2024	800 IU [20 μg] daily for those older than 70 years	Recommendation against routine screening	Serum 25(OH)D	NA	NA	NA
	ESCEO [56]	2022	800–1000 IU daily	Not provided. Supplementation recommended in persons at increased risk of deficiency	Serum 25(OH)D concentration	Not provided	Not provided	Deficiency: <25 or 30 nmol/L
	BBC [3]	2020	800–1000 IU daily	Postmenopausal women with at least 1 major risk factor for osteoporosis who undergo further assessment for osteoporosis	Serum 25(OH)D (accurate and standardized method)	Women age ≥ 65 years who undergo assessment for osteoporosis	Monitoring in those on supplements; re-screening every 2 years if non-treated	Treatment target > 50 nmol/L
After bariatric surgery	BOMSS [74]	2020	2000–4000 IU daily, adjusted as per monitoring	Adults undergoing bariatric surgery	Serum 25(OH)D	Pre-surgery	3, 6 and 12 months in the first year and at least annually	Serum 25(OH)D > 75 nmol/L considered sufficient
	ES [73]	2010	Not provided	Adults undergoing bariatric surgery	Serum 25(OH)D	Pre-surgery	Every 6 months	Serum 25(OH)D > 75 nmol/L considered optimal
After fragility fracture	EULAR/EFFORT [96]	2017	800 IU daily	Patients older than 50 years with a fragility fracture	Not provided	When clinically indicated	Not provided	Not provided

ACOG: American College of Obstetricians and Gynecologists; AGA: American Gastroenterology Association; BBC: Belgian Bone Club; BOMSS: British Obesity and Metabolic Surgery Society; BSG: British Society for Gastroenterology; EFFORT: European Federation of National Associations of Orthopaedics and Traumatology; EMAS: Menopause and Andropause Society; ES: Endocrine Society; ESCEO: European Society of Clinical and Economical Aspects of Osteoporosis, Osteoarthritis and Musculoskeletal diseases; ESE: European Society for Endocrinology; EULAR: European League Against Rheumatism; IFCC: International Federation of Clinical Chemistry; KDIGO: Kidney Disease Improving Outcomes; NA: not applicable; USPSTF: U.S. Preventive Services Task Force; and RDI: Recommended Daily Intake. To convert 25(OH)D levels from nmol/L to ng/mL, divide by 2.496.

## 3. How to Evaluate Vitamin D Status?

### 3.1. The Issues with Serum 25(OH)D?

A major hurdle remains the lack of a good estimate of vitamin D bioavailability [112,113,114]. Although serum 25(OH)D concentration (i.e., the sum of 25(OH)D2 and 25(OH)D3) is still recommended as the biomarker of choice to estimate vitamin D stores, there are limitations related to both analytical aspects and the interpretation of serum 25(OH)D concentrations [15,115]. For instance, while, in normal individuals, the accuracy and reproducibility of automated assays have improved, this is not necessarily so in specific populations such as children, pregnant women, and patients with chronic renal or hepatic disease [39,115]. Another important barrier to implement efficient screening programs and define optimal diagnostic cut-off levels is the rather poor agreement between different vitamin D measurement methods. This is one of the reasons for the disagreement among experts and scientific societies regarding the optimal 25(OH)D level for sufficiency, leading to ongoing debates (also see Table 2). In routine practice, serum 25(OH)D levels are measured using commercial immunoassays. Thus, before being able to define clinical status based on 25(OH)D concentrations, it is crucial to ensure standardized results across all assays used for 25(OH)D determination. In other words, a comprehensive standardization of all available assays is necessary to ensure consistent cut-off values. To address this issue, the Vitamin D Standardization Program was established in 2010 as an international collaborative effort involving various institutions, including the National Institute of Standards and Technology (NIST), the Centres for Disease Control and Prevention (CDC), Ghent University, the International Federation of Clinical Chemistry, and regional scientific associations. Currently, three reference methods procedures (RMPs) based on ID-LC-MS/MS (NIST, CDC, and Ghent University) have been developed and recognized by the Joint Committee for Traceability in Laboratory Medicine (JCTLM). The CDC has also initiated an international Vitamin D Standardization Certification Program for research, clinical laboratories, and manufactured kits. These efforts have significantly improved the standardization of 25(OH)D assays, although some challenges remain. These include deviations dependent on patients or matrices, particularly (but not only) related to the vitamin D-binding protein (DBP) concentration and/or polymorphism (e.g., pregnant women, patients in intensive care, patients on haemodialysis, patients with osteoporosis, patients with liver failure), matrix effects, heterophilic antibody interferences, variations in cross-reactivity with 25(OH)D2, significant cross-reactivity with 24,25(OH)_2_D, and incomplete separation of the C3-epimer of 25(OH)D from 25(OH)D in certain LC-MS/MS methods [114].

Also, it must be considered that, as a lipophilic steroid, serum vitamin D is strongly bound to DBP and more loosely to albumin with only less than 0.1% circulating freely. According to the free hormone hypothesis, only the latter two fractions are available for biological action and metabolism, and indeed, there are several reports on how these (calculated) concentrations of bioavailable and free vitamin D better associate with, e.g., markers of bone metabolism [112,114,116,117,118,119]. As most commercially available assays measure total 25(OH)D levels, their use might lead to misconception of vitamin D status in conditions affecting production, clearance, and binding affinity of vitamin D and the DBP such as proteinuric renal or hepatic disease [112,120,121]. Also, there are several genetic variants identified as important determinants of vitamin D metabolism and serum 25(OH)D levels, probably affecting vitamin D bioavailability [115]. Although there is at least one commercial immunoassay to measure free vitamin D available, it lacks validation [114,115]. Alternatively, calculators for free vitamin D can be used; however, the proposed equations are not specifically designed for estimating free or bioavailable vitamin D levels, especially not in diseased populations [15,112,115,122,123]. Moreover, the choice of DBP assay for the calculation of free 25(OH)D is important as there are discrepancies between different analytic DBP methods, with monoclonal assays apparently sensitive to genetic DBP polymorphisms [24,124,125,126], a problem which could be overcome using LC-MS/MS [127]. For now, however, given all these uncertainties and lack of validation, clinicians will continue to have to rely on measures of total rather that free serum 25(OH)D levels.

### 3.2. The Measurement and Clinical Interest of the 24,25(OH)_2_D Metabolite

The amount of circulating 24,25(OH)_2_D depends on the amount of 25(OH)D and the activity of 24-hydroxylase. Expression of *CYP24A1* is upregulated by 1α,25(OH)_2_D and FGF23, downregulated by PTH, and partly regulated by VDR activity. When vitamin D reserves are insufficient, the enzyme is inactive, whereas it starts degrading vitamin D when stores start to replenish [58]. Therefore, the calculation of the vitamin D metabolite ratio (VMR, i.e., the ratio of 24,25(OH)_2_D to 25(OH)D) has been considered by different authors as a better indicator of vitamin D sufficiency/deficiency than 25(OH)D alone [15,115]. For instance, the VMR is not affected by race nor by DBP concentration [128]. Measurement of 24,25(OH)_2_D is also of primary importance in the detection of mutations in *CYP24A1*, leading to its partial or total decrease in activity. Loss of function mutations are associated with a clinical phenotype characterized by low PTH levels, increased 1,25(OH)_2_D, hypercalcemia, hypercalciuria, and/or kidney stones. Biallelic mutations of *CYPA24A1* can lead to idiopathic infantile hypercalcemia (IIH) [15].

From an analytical perspective, the measurement of 24,25(OH)2D can currently be only performed with LC-MS/MS methods. Such methods present high sensitivity and specificity but are not available in all clinical labs. On the other hand, they allow for the simultaneous quantitation of 25(OH)D and 24,25(OH)_2_D, allowing an easy calculation of the VMR. Good news is that a candidate RMP based on ID-LC-MS/MS for the determination 24,25(OH)_2_D has been developed by the NIST and recognized by JCTLM. This method was recently used to assign values for 24,25(OH)_2_D3 in two standard reference materials (SRM972a and SRM2971), and DEQAS is offering an accuracy-based external quality assessment scheme for 24,25(OH)_2_D.

### 3.3. The Measurement and Clinical Relevance of 1,25(OH)2D

1,25(OH)_2_D is the most active form of vitamin D. However, its measurement does not represent vitamin D stores and should be limited to diagnosing certain rare but serious disorders of calcium, phosphate, and bone metabolism. These include conditions such as hypocalcaemia or hypercalcemia not caused by parathyroid disorders, various forms of hypophosphatemia, and unexplained osteomalacia or rickets. This metabolite should neither be measured in the follow-up of patients suffering from chronic kidney disease or patients on haemodialysis [110,129]. The enzyme that converts 25(OH)D into 1,25(OH)_2_D is tightly regulated, and 1,25(OH)_2_D circulates in picomolar concentrations, i.e., 1000 times lower than 25(OH)D. 1,25(OH)_2_D is generally measured with immunoassays, either manual or automated. These latter appear to perform better regarding cross-reactivity and sensitivity [115]. Very few labs have developed LC-MS/MS methods to measure 1,25(OH)_2_D, and such methods need a complex sample preparation to detect the very low concentrations of the analyte with enough specificity. There is so far no reference method for measuring 1,25 (OH)2D, and the standardization of assays is not yet achieved, leading to large discrepancies between the assays.

## 4. Challenges and Perspectives

The task of appraising vitamin D status and understanding its bioavailability poses ongoing challenges within the realm of healthcare. Recognizing the intricacies involved, it becomes evident that a one-size-fits-all approach to screening for deficiencies or disturbances in vitamin D metabolism is not practical. Instead, a judicious strategy involves targeted screenings directed at specific populations, acknowledging the varied factors that contribute to vitamin D dynamics.

In proposing periodic screening in specific populations, such as on an annual basis, we aim to strike a balance between vigilance and practicality. This approach recognizes the dynamic nature of vitamin D metabolism and allows for timely interventions in populations where the risk of deficiency or metabolic disruptions is higher. However, there is a dearth of evidence with respect to the optimal start of screening, timing of screening (i.e., not during or shortly after excessive sun exposure during summer or after vacation), and monitoring frequency, especially in populations at the highest risk such as nursing home residents and persons with inflammatory bowel disease, malabsorption syndromes including those after bariatric surgery, and other conditions associated with vitamin D deficiency or increased fracture risk.

When it comes to assessing vitamin D status through serum 25(OH)D levels, the utilization of immunoassays is a viable option, although one must remain cognizant of their inherent limitations. These limitations may include potential variations in accuracy and precision, specifically in some populations. Despite these constraints, immunoassays provide a reasonable and convenient method for routine clinical evaluations.

However, for a more granular and nuanced understanding of vitamin D status, especially in complex clinical scenarios, the adoption of LC-MS/MS becomes imperative. Indeed, LC-MS/MS offers a higher level of accuracy and precision, enabling a more detailed analysis of vitamin D metabolites. This advanced technique is particularly valuable in situations where subtle variations in vitamin D levels may have clinical significance, providing healthcare practitioners with a more comprehensive tool for diagnostic decision making. However, for many of the known vitamin D metabolites, their clinical relevance needs to be further demonstrated.

In conclusion, the multifaceted nature of assessing vitamin D status necessitates a thoughtful and tailored approach. By considering targeted screenings and utilizing advanced methodologies like LC-MS/MS when warranted, healthcare professionals can enhance their ability to understand and address vitamin D-related issues in diverse clinical contexts.

## 5. Summary

The appraisal of vitamin D status and bioavailability remains a challenge. Screening for deficiencies or disturbances in vitamin D metabolism should not be performed universally, but in targeted populations. Although not limitative, we propose periodical (e.g., yearly) screening in individuals with osteoporosis or at risk for fragility fractures, in those on substitution to monitor compliance and adapt dosing, and in populations at increased risk of vitamin D deficiency such as people with limited sun exposure, patients with intestinal malabsorption, or those using medications affecting vitamin D metabolism. In general, an assessment of vitamin D status by serum 25(OH)D levels can be performed using available immunoassays, keeping their limitations in mind. However, for a more detailed appraisal of vitamin D status, LC-MS/MS is needed, which will also allow for a more profound analysis, e.g., including the VMR.
